# Anti-Tumor Effects of Ketogenic Diets in Mice: A Meta-Analysis

**DOI:** 10.1371/journal.pone.0155050

**Published:** 2016-05-09

**Authors:** Rainer J. Klement, Colin E. Champ, Christoph Otto, Ulrike Kämmerer

**Affiliations:** 1 Department of Radiotherapy and Radiation Oncology, Leopoldina Hospital Schweinfurt, Schweinfurt, Germany; 2 Department of Radiation Oncology, University of Pittsburgh Medical Center, Pittsburgh, Pennsylvania, United States of America; 3 Department of General, Visceral, Vascular and Pediatric Surgery, University Hospital of Würzburg, Würzburg, Germany; 4 Department of Obstetrics and Gynaecology, University Hospital of Würzburg, Würzburg, Germany; Columbia University, UNITED STATES

## Abstract

**Background:**

Currently ketogenic diets (KDs) are hyped as an anti-tumor intervention aimed at exploiting the metabolic abnormalities of cancer cells. However, while data in humans is sparse, translation of murine tumor models to the clinic is further hampered by small sample sizes, heterogeneous settings and mixed results concerning tumor growth retardation. The aim was therefore to synthesize the evidence for a growth inhibiting effect of KDs when used as a monotherapy in mice.

**Methods:**

We conducted a Bayesian random effects meta-analysis on all studies assessing the survival (defined as the time to reach a pre-defined endpoint such as tumor volume) of mice on an unrestricted KD compared to a high carbohydrate standard diet (SD). For 12 studies meeting the inclusion criteria either a mean survival time ratio (MR) or hazard ratio (HR) between the KD and SD groups could be obtained. The posterior estimates for the MR and HR averaged over four priors on the between-study heterogeneity τ^2^ were MR = 0.85 (95% highest posterior density interval (HPDI) = [0.73, 0.97]) and HR = 0.55 (95% HPDI = [0.26, 0.87]), indicating a significant overall benefit of the KD in terms of prolonged mean survival times and reduced hazard rate. All studies that used a brain tumor model also chose a late starting point for the KD (at least one day after tumor initiation) which accounted for 26% of the heterogeneity. In this subgroup the KD was less effective (MR = 0.89, 95% HPDI = [0.76, 1.04]).

**Conclusions:**

There was an overall tumor growth delaying effect of unrestricted KDs in mice. Future experiments should aim at differentiating the effects of KD timing versus tumor location, since external evidence is currently consistent with an influence of both of these factors.

## Introduction

While the first study assessing the effect of diet on cancer dates back to 1909 [1], there has recently been a surge of attention regarding the metabolic hallmarks of cancer and the possibility to influence them through dietary manipulation. Dietary restriction, either of overall energy consumption [1–4] or defined nutrients such as proteins [5,6] or carbohydrates [7,8], has become the prime example of a dietary intervention influencing key pathways, growth and metabolism of cancer. Such studies are difficult to perform in cancer patients, thus murine tumor models have served as an essential tool to study the effect of dietary changes on these pathways. In mice, dietary restriction has been shown to protect against the initiation of cancer and to slow tumor growth after the manifestation of cancer [1,9]. Mechanistically, this has been linked to decreased levels of glucose, insulin and insulin-like growth factors [10,11]. The most potent anti-tumor effects have been described for fasting [12] which additionally leads to a rapid increase in circulating ketone bodies in both men [13] and mice [14]. Ketogenic diets (KDs), on the other hand, are fasting-mimicking diets that also lead to an increase in ketone bodies without the need to restrict energy intake—a clear advantage in the cancer setting [15,16]. A KD is typically composed of at least 75% fat with a maximum 10% of energy from carbohydrate sources, corresponding to a ketogenic ratio of about 2:1. The ketogenic ratio is defined as the weight percentage of fat in the diet divided by the combined weight percentage of protein and carbohydrate [17].

KDs have shown anti-tumor potential in many, but not all mouse studies. The reason for this discrepancy is not yet clear. In some studies, calorie restriction was required to elicit a potent anti-tumor effect [18]. It has been argued that a lack of decreasing blood glucose levels with *ad libitum* feeding would explain the lack of efficiency without caloric restriction. Indeed, most murine tumor models report no significant decreases in blood glucose levels, and while some studies do reveal a decrease [19–21], others report lower insulin levels compared to controls despite unchanged or even elevated glucose levels [22,23].

In addition, a collective interpretation of murine tumor models is hampered by the large variety of experiment setups and the small number of animals used in most studies. Thus there remains some uncertainty concerning the anti-tumor effects of a KD in current preclinical models. We therefore conducted a systematic review of the literature to ascertain the effects of a KD on tumor growth, and to determine possible factors that may account for heterogeneity in response to the KD.

## Materials and Methods

### Selection criteria

The inclusion criteria for this meta-analysis were defined a priori as follows:

1. Studies investigating tumor growth in a murine cancer model.
2. Studies testing the effects of an **unrestricted** KD with a ketogenic ratio of at least 2:1 on tumor growth in comparison to a control standard diet (SD) with at least 50% energy content from CHO **without** additional treatment.
3. Endpoint defined as reaching a pre-defined tumor volume or other sign of disease progression with no termination of the experiment at a pre-defined time interval.
4. Conduction of a survival analysis with the specified endpoint, so that in principle either a hazard ratio (HR) or a mean survival time ratio (MR) between the KD and the control diet groups could be calculated.

Studies not fulfilling all of the above four inclusion criteria were excluded from the analysis. No registered protocol existed for this study.

### Search strategy

Potentially relevant studies were searched January 5, 2016 in the PubMed database using the search terms “ketogenic diet” AND “cancer”. References of selected articles and review articles on this subject were searched for additional studies.

### Data extraction

Data from each study and risk of bias were extracted independently by two authors (RJK and CEC) using a preset form. In case of discrepancies between extracted data, consent was found by discussion between these two reviewers. For each study, we recorded the year it was published, the first author’s name, the tumor model used, the number of animals in each diet group, the time when the diet intervention was started (prior to/at the same day of/after tumor implantation), the ketogenic ratio of the chow and whether body weight under the KD increased, decreased, or remained unchanged compared to the control regimen (Table 1). Since the time points at which ketone body or glucose levels were evaluated in each study differed substantially, we decided to simply record whether there was a statistically significant difference (p<0.05) in these blood parameters at least once during the intervention. Furthermore, the principle outcome measures MR and HR were extracted. Both were defined such that ratios less than one indicated a beneficial effect of the KD. The HR is equivalent to the odds of dying first and thus related to the probability *P* that a mouse from the KD group dies before a mouse from the control group according to *P* = HR/(1+HR) [24]. 95% confidence intervals (CIs) for the HR and standard errors (SE) of the mean survival times were also extracted; from the latter 95% CIs for the MR were derived.

**Table 1:** Studies fulfilling all inclusion criteria for this meta-analysis: General data.

Publication year	Study	Tumor model	Model details	Location	N_KD_+N_SD_	Diet initiation	Ketogenic ratio	Ketosis	Glycemia	Body weight	Comment
2007	Zhou	S	CT-2A brain tumor i.c., C57BL/6J mice	Brain	9+7	after	4:1	+	0	0	This study had two separate experiments. High risk of reporting bias (no HR/MR given).
		X	U87 glioma s.c., C57BL/6J mice	Brain	7+11	after	4:1	+	0	0	
2008	Freedland	X	LNCaP prostate s.c.	s.c.	25+25	prior	2.1:1	+	+	0	SD defined as the Western diet. KD mice heavier than controls at tumor implantation, but this was accounted for in HR computation.
2008	Otto	X	23132/87 gastric cancer s.c., NMRI mice	s.c.	12+12	day 0	2.7:1	+	0	0	High risk of selection, performance and other bias (KD mice lighter than controls at tumor implantation; individual who performed the experiments also analyzed the data; conflicts of interest).
2009	Mavropoulos	X	LAPC-4 prostate s.c., SCID mice	s.c.	48+41	prior	2.1:1	+	0	0	SD defined as the Western diet. High risk of selection bias (KD mice heavier than controls at tumor implantation).
2010	Stafford	S	GL261 glioma i.c., C57BL/6 mice	Brain	5+5	after	6:1	+	NA	NA	High risk of reporting bias (no body weight trends reported)
2011	Maurer	X	LNT-229 glioma i.c., athymic Foxn1nu mice	Brain	12+12	after	2.7:1	+	0	0	High risk of reporting bias (no HR/MR given). Four mice in the SD group and two in the KD group were censored and not considered for mean survival time computation
2012	Abdelwahab	S	GL261 glioma i.c., C57BL/6 mice	Brain	20+19	after	4:1	+	-	0	One mouse in the KD group was cured and not considered for mean survival time computation. High risk of performance bias (individual who performed the experiments also analyzed the data; conflicts of interest).
2013	Poff	S	VM-M3 metastatic cancer, s.c., VM/Dk mice	s.c.	8+13	day 0	4:1	0	-	-	Ketone bodies on KD elevated, but not significantly. High risk of performance bias (individual who performed the experiments also analyzed the data).
2014	Rieger	X	U87MG glioma cells i.c., athymic Foxn1nu mice	Brain	8+8	after	3.1:1	+	0	0	High risk of reporting and other bias (no HR/MR given; conflicts of interest).
2015	Hao	X	HCT116 colorectal s.c., BALBc/J SCID male	s.c.	24+12	day 0	3:1	+	0	0	Two KDs used (MKD and LKD); both groups pooled together.
2015	Dang	S	Spontaneous murine medulloblastoma, genetically engineered Ptch1+/- Trp53-/- mice on C57Bl/6:129SV 0background	Brain	4+4	after	4:1	+	NA	+	High risk of reporting, performance and other forms of bias (no HR/MR given; individual who conducted the experiment also analyzed the data; no ketone body measurements reported).
2015	Martuscello	X	Patient-derived L0 glioblastoma cells i.c., NOD/SCID mice	Brain	10+11	after	6:1	+	-	-	Two ketogenic diets used (KD and sHFLC) but only KD considered due to its high ketogenic ratio. High risk of selection, reporting and other forms of bias (time from tumor implantation until KD initiation differed by up to 4 days; no HR/MR given).

Diet initiation refers to “day 0” which is the day of tumor implantation. S: syngeneic; X: xenogeneic. Ketosis and glycemia are coded such that 0 indicates that no statistically significant differences between both groups were found at any measurement (p>0.05), while the + and - signs indicate that there was at least one measurement in which ketosis or blood glucose levels in the treatment group were significantly higher (+) or lower (-), respectively, compared to the control mice.

If no mean survival times or uncertainty estimates were provided in the article, the corresponding study author was contacted by one of us (RJK) to obtain this information. One study [20] only reported a p-value based on a *t*-test comparison of the mean survival times. Although this assumes that the individual survival times are normally distributed (which is usually not the case and questionable even without censoring as in this study), we stayed consistent with this assumption and used this p-value to estimate a SE for the mean survival time differences according to the guidelines of Altman & Bland [25]; finally this SE was divided by $\sqrt{2}$ to obtain the SE of the individual mean survival times in both groups under the assumption that they would be equal.

Risk of bias was assessed by using the Systematic Review Centre for Laboratory animal Experimentation (SYRCLE) tool which consists of 10 items for which judged based on a number of signaling questions [26]. It was decided to test the sensitivity of the results to withholding studies with high risk of bias.

Finally, one of us (UK) extracted approximate blood concentrations of ketone bodies and glucose from figures and data, which was possible for 10 studies. As crude estimates, these were treated with care and only used to get an idea of the range of ketosis and blood glucose levels in the mice.

### Statistical analysis

We conducted a Bayesian meta-analysis. Compared to the classical approach this has several advantages such as obtaining direct probability distributions for the parameters of interest, naturally accounting for the full uncertainty in the parameters and allowing each individual study “borrowing strength”, i.e., utilizing information from all other studies for estimating the “true” study treatment effect [27,28]. To compare the effects of a KD with a SD, the MR was defined as the primary and the HR as the secondary outcome of interest. The MR is typically more appropriate than the HR for these type of studies in which the specified endpoint is eventually reached by all animals [24]. All ratios were transformed to the natural logarithmic scale prior to analysis.

We anticipated different, yet similar, effects of the KD intervention between the studies, so that a random effects model was used [28]. A normal likelihood for the individual study observations was assumed [27–29]:

$$y_{i}\sim N\left(\theta_{i},s_{i}^{2}\right)$$ (1)

Here *y*_*i*_ and *s*_*i*_ denote the outcome [ln(MR) or ln(HR)] and its SE in the *i*th study, and the true study effects *θ*_*i*_ are assumed to be exchangeable [28] and drawn from an underlying distribution given by

$$\theta_{i}\sim N\left(\mu ,\tau^{2}\right)$$ (2)

Heterogeneity was assessed by the between study variance τ^2^ which was modeled using four different prior distributions [27,29]: (i) a prior for τ uniform on [0,2]; (ii) a half-normal prior for τ with standard deviation 0.25, corresponding to an anticipated “upper” value for τ of 0.49; (iii) a Gamma(0.001,0.001) prior on 1/τ^2^, which is close to being uniform on log(*τ*); (iv) DuMouchel’s prior $P\left(\tau \right)=\frac{s_{0}}{{\left(s_{0}+\tau \right)}^{2}}$ with $s_{0}^{2}=K/\left(\sum_{i=1}^{K}s_{i}^{-2}\right)$ being the harmonic mean of the *K* individual study variances $s_{i}^{2}$. Using four different priors for τ probes the sensitivity of the results to different *a priori* assumptions about the between-study heterogeneity.

Finally, Bayesian meta-regression [27,29] was conducted to determine the source of heterogeneity. Due to the small number of studies only univariate analysis was conducted:

$$\theta_{i}\sim N\left(\mu +\beta x_{i},\tau^{2}\right)$$ (3)

Here, *x*_*i*_ is the covariate (also called moderator) for study *i* and *β* its regression coefficient. Because ketosis, blood glucose and body weight trends were too uniform across the studies, we decided to investigate the impact of the publication year, the tumor model (syngeneic/xenogeneic), the tumor location (intracranial/subcutaneous), the ketogenic ratio of the KD and time of diet initiation as moderators of the MR and HR in subgroup analysis.

All analysis was conducted with R version 3.1.3 with the BRugs package and OpenBugs version 3.2.2. Two Markov chains were individually initialized and the first 10000 Markov chain Monte Carlo samples discarded. For the next 25000 iterations every fifth sample was kept to obtain the posterior parameter distribution for the parameters of interest. The median was taken as the parameter estimate and parameters considered “significant” when their 95% highest posterior density interval (HPDI) excluded zero.

## Results

**Fig 1:**
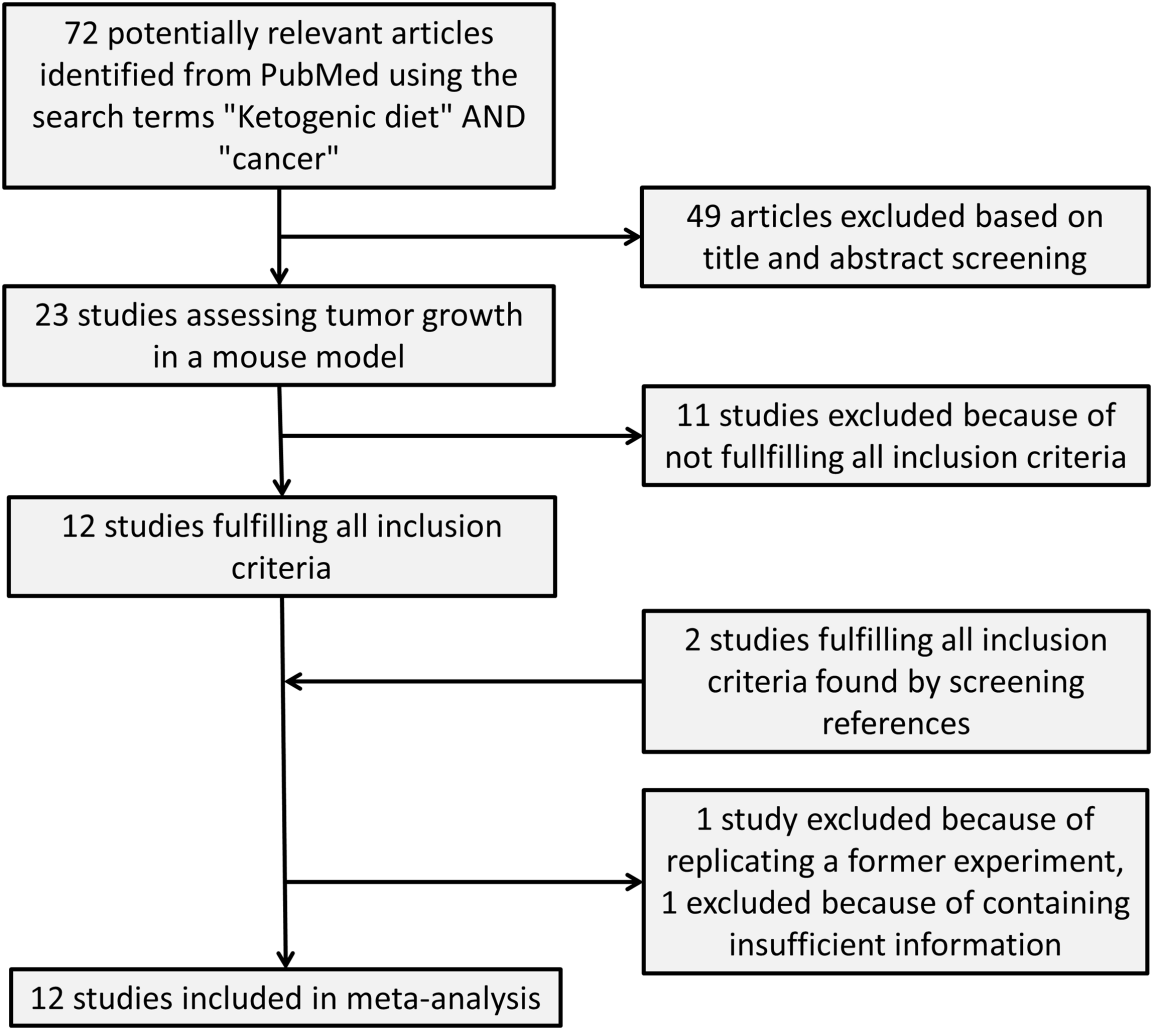
Flow chart of the study selection procedure.

The PubMed search for “ketogenic diet” AND “cancer” resulted in a total of 72 articles of which 23 were studies investigating the effects of a KD on tumor growth in a mouse model (Fig 1). From these 23 studies, seven were excluded because they conducted no survival analysis [30–36], three were excluded since they terminated the experiments after a pre-defined time interval [37–39] and one study was excluded because it had no control diet with >50% energy from CHO [40]. Finally, we excluded the study of Poff et al. [41] published in 2015 since it was essentially a replication of an earlier study of these authors with the same tumor model and same mean survival time of the SD group. The remaining 11 studies fulfilled all inclusion criteria and were considered for data extraction [7,18,20–23,42–46]. The study of Zhou et al. [18] included two different tumor models which were evaluated separately. Two more studies fulfilling the inclusion criteria were found by searching the references of review articles on this subject [19,47]. One of these two was a complementary mouse study to a clinical trial involving glioblastoma patients [47]. An additional search for (“Low carbohydrate diet” OR “Atkins diet”) AND “cancer” did not reveal any further studies fulfilling the inclusion criteria.

We were able to obtain sufficient data to compute either a MR or a HR from 12 of the 13 selected studies. The study supplying insufficient information was excluded (Fig 1). It used a castrated prostate cancer xenograft model in which a significantly beneficial effect of a KD compared to a Western-type diet was reported [7].

The general design and results of the included 12 studies is given in Table 1, while Table 2 provides results concerning the survival outcomes. Only two studies reported a HR [22,23]; for six others we were able to retrieve the complete survival time data either from the authors [19,42–44,47] or by reading them off the Kaplan-Meier plot [46], and computed HRs and 95% CIs from the Cox proportional hazards model. From these complete datasets we also derived mean survival times with their SE. Further mean survival times were either extracted directly from the studies [20,45] or provided by the study authors [18,21]. This resulted in a sample of 11 studies (with 12 experiments) measuring a MR and 7 studies (8 experiments) measuring a HR.

**Table 2:** Studies fulfilling all inclusion criteria for this meta-analysis: Outcome data.

Publication year	Study	T_KD_ [days]	T_SD_ [days]	MR	MR 95% CI	HR	HR 95% CI	Data source
2007	Zhou	19.7±0.9	16.7±1.4	0.85	[0.69,1.01]	NA	NA	Mean survival times provided by author
		18.7±0.9	22.5±1.8	1.20	[0.98,1.42]	NA	NA	
2008	Freedland	NA	NA	NA	NA	0.48	[0.27,0.86]	Publication
2008	Otto	34.2±2.5	23.3±1.1	0.68	[0.57,0.80]	0.16	[0.05,0.53]	Individual survival times provided by author
2009	Mavropoulos	NA	NA	NA	NA	0.59	[0.37,0.93]	Publication
2010	Stafford	24±1.1	19±0.7	0.79	[0.70,0.89]	0.07	[0.01,0.63]	Individual survival times provided by author
2011	Maurer	82.4±1.2	94.9±1.3	1.15	[0.89,1.49]	1.65	[0.65,4.21]	Individual survival times provided by author
2012	Abdelwahab	28.8±1.5	23.3±1.1	0.81	[0.70,0.92]	0.35	[0.17,0.71]	Individual survival times provided by author
2013	Poff	48.9±4.4	31.2±4.4	0.64	[0.43,0.85]	NA	NA	T_KD_ and T_SD_ taken from publication, standard errors computed from p-value (see text for details)
2014	Rieger	35.6±0.7	33.9±1.6	0.95	[0.85,1.05]	0.79	[0.28,2.24]	Individual survival times provided by author
2015	Hao	34.5±10.1	24.8±3.1	0.72	[0.27,1.17]	NA	NA	Publication
2015	Dang	17.8±0.5	16.3±2.3	0.92	[0.66,1.17]	1.43	[0.82,6.30]	Publication; individual survival times read off Kaplan-Meier plot
2015	Martuscello	56±4.2	38±1.0	0.68	[0.57,0.78]	NA	NA	Mean survival times provided by author

T_KD_ and T_SD_ denote the mean survival times in the KD and SD groups, respective, and are given with their SE. These SE have been used to compute the 95% CI.

Not reporting MR and HR despite conducting a survival analysis was considered as evidence for reporting bias. By retrieving these measures from the study authors we eliminated the influence of this bias on the cumulative evidence. However, several other forms of bias were identified in all but one study (Table 1), and not directly identified risk of bias was mostly considered unclear since several aspects of methodology such as generation of the randomization sequence, allocation concealment or random outcome assessment [26] were not reported. Overall, the risk of bias was judged as high but similar enough between studies to not account for it in the analyses. An exception was possible bias due to financial conflicts of interest reported by one or more study authors which we accounted for by conducting a sensitivity analysis with the corresponding studies withheld.

When all studies were pooled together, a total of 192 mice were treated with a KD and 180 mice fed a SD. Mice receiving a KD had higher ketone body concentrations which was significant in all studies but one [20] (mean concentrations extracted from 10 studies 1.6±0.4 mM versus 0.3±0.1 mM). In most studies there were no significant differences in blood glucose levels between both treatment groups, but on average concentrations on the KD tended to be lower (7.0±1.0 mM versus 8.5±1.0 mM).

Eight of the 13 experiments found a significantly longer survival for mice receiving a KD compared to a SD. The result of the meta-analysis for the overall effect of a KD on the MR and HR is shown in Tables 3 and 4. The posterior estimates for the MR and HR averaged over all four priors on τ^2^ were MR = 0.85 (95% HPDI = [0.73, 0.97]) and HR = 0.55 (95% HPDI = [0.26, 0.87]). Thus there was a significant overall benefit of the KD in terms of prolonged mean survival times and reduced odds of dying first. The effect measure estimates were not sensitive to the type of prior used for the between-study variance. The estimate of τ^2^, however, was highly sensitive to its prior in the meta-analysis when HR was used as the outcome. This probably reflects the greater uncertainty associated with the small number of studies. With MR as the effect measure, estimates of τ^2^ were more uniform and reasonable, but the 95% HPDI supported both very small and substantial heterogeneity [29].

**Table 3:** Results of the Bayesian meta-analyses for the mean survival time ratio (MR) investigating four different priors for the between-study variance.

Prior on *τ*	Uniform prior	Half-normal prior	Inverse gamma prior	DuMouchel prior
**Overall effect exp(*μ*)**				
Prior distribution	*μ*~N(0,100)	*μ*~N(0,100)	*μ*~N(0,100)	*μ*~N(0,100)
Posterior median	0.85	0.85	0.85	0.85
Standard deviation	0.06	0.06	0.06	0.06
95% HPDI	[0.72,0.98]	[0.74,0.97]	[0.73,0.97]	[0.74,0.96]
**Between-study variance *τ***^**2**^				
Prior distribution	*τ~*U(0,2)	*τ~*HN(0,0.25)	$\tau^{-2}\sim G\left(0.001,0.001\right)$	$P\left(\tau \right)=\frac{s_{0}}{{\left(s_{0}+\tau \right)}^{2}}$
Posterior median	0.0388	0.0334	0.0315	0.0290
Standard deviation	0.0367	0.0266	0.0300	0.0260
95% HPDI	[0.0106,0.1446]	[0.0099,0.1078]	[0.0086,0.1165]	[0.0082,0.1022]

**Table 4:** Similar to Table 3, but for the hazard ratio (HR) as the effect measure.

Prior on *τ*	Uniform prior	Half-normal prior	Inverse gamma prior	DuMouchel prior
**Overall effect exp(*μ*)**				
Prior distribution	*μ*~N(0,100)	*μ*~N(0,100)	*μ*~N(0,100)	*μ*~N(0,100)
Posterior median	0.54	0.55	0.55	0.55
Standard deviation	0.20	0.11	0.17	0.16
95% HPDI	[0.25,1.04]	[0.38,0.80]	[0.27,0.87]	[0.30,0.92]
**Between-study variance *τ***^**2**^				
Prior distribution	*τ~*U(0,2)	*τ~*HN(0,0.25)	$\tau^{-2}\sim G\left(0.001,0.001\right)$	$P\left(\tau \right)=\frac{s_{0}}{{\left(s_{0}+\tau \right)}^{2}}$
Posterior median	0.49	0.0649	0.1914	0.1886
Standard deviation	0.7251	0.1111	0.658	0.5987
95% HPDI	[0.0198,2.842]	[0.0002,0.4019]	[0.0012,2.076]	[0.0004,1.822]

The overall protective effect was still apparent after excluding three studies with high risk of bias due to financial conflicts of interest [19,42,47], but the 95% HPDI now included 1 (MR = 0.87, 95% HPDI = [0.70,1.04]). Also, excluding the study by Dang et al. [46] from which we extracted the individual survival times out of the Kaplan-Meier plot did not change the positive treatment effect estimate but slightly increased its uncertainty (MR = 0.84, 95% HPDI = [0.70,1.01]).

**Fig 2:**
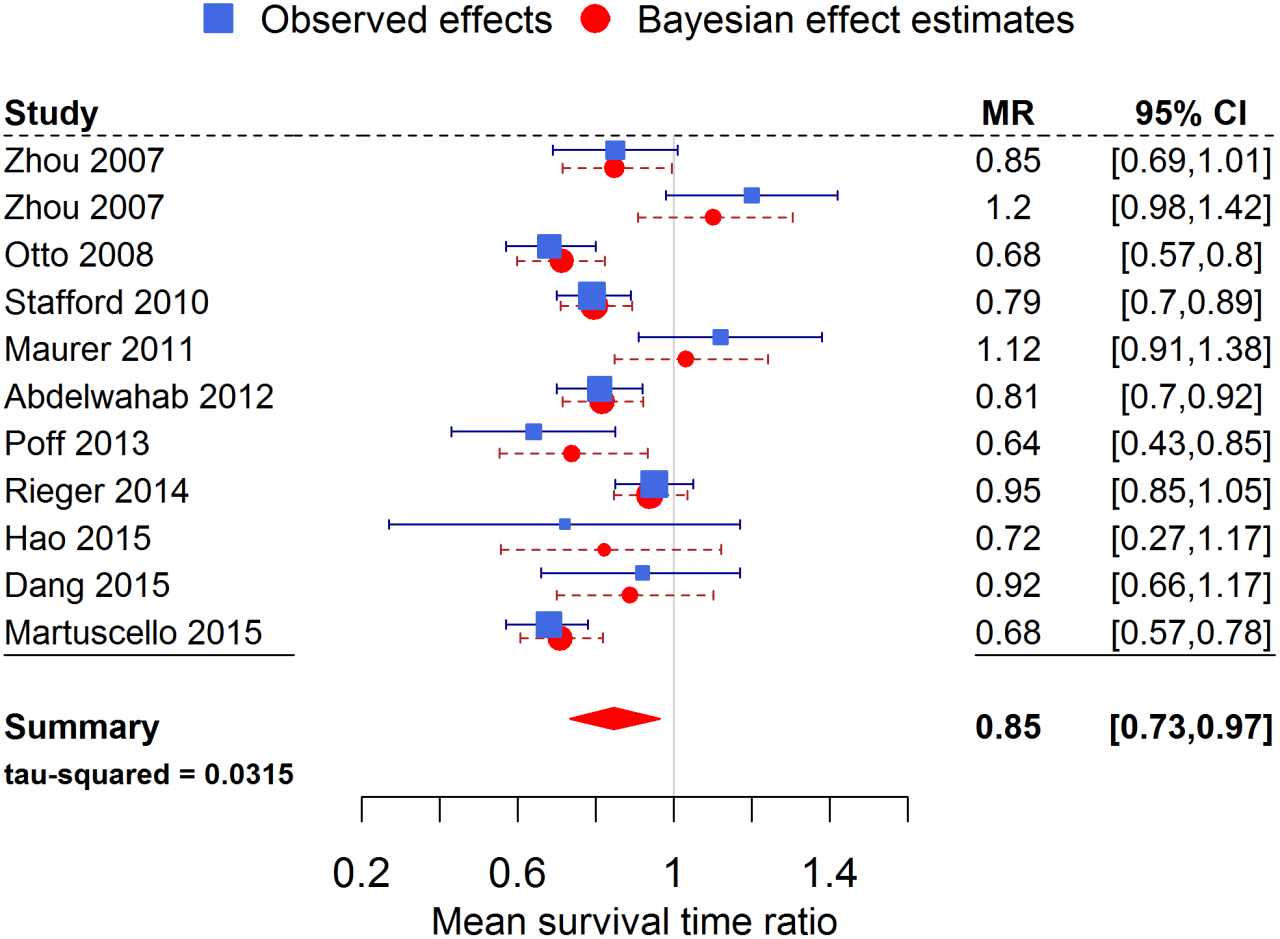
Forest plot of the meta-analysis for the mean survival time ratio. Values less than 1 indicated a beneficial effect of the KD. The observed effects Eq (1) are the effects extracted from the individual studies, while the Bayesian effect estimates Eq (2) represent the true study effects and are influenced by all the other studies.

**Fig 3:**
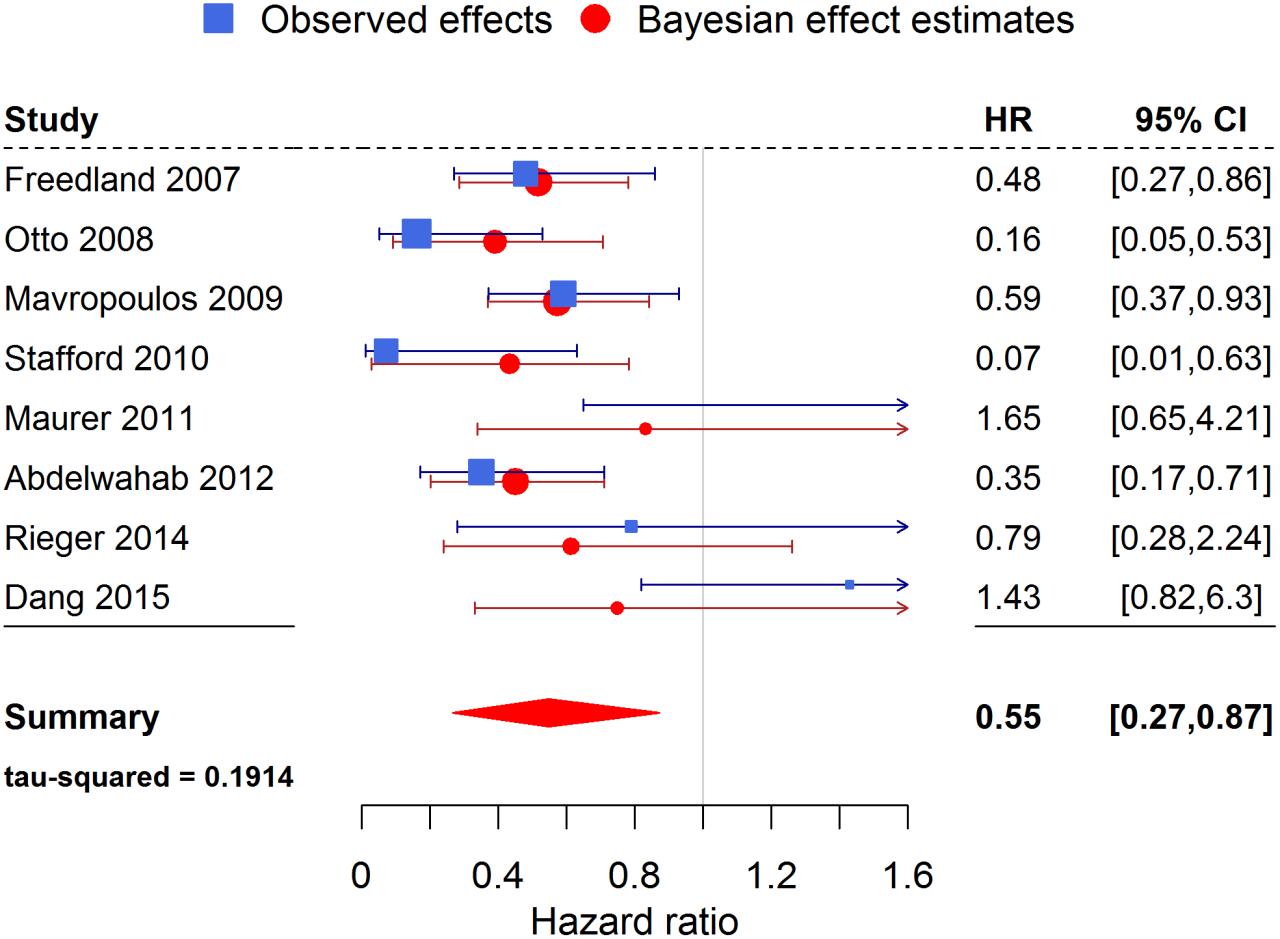
Forest plot of the meta-analysis for the hazard ratio. Values less than 1 indicated a beneficial effect of the KD.

Figs 2 and 3 show a forest plot for MR and HR, respectively, as the effect measure using the inverse gamma prior on τ^2^. Note how the Bayesian estimates of the true study effects of each trial are shifted towards the overall pooled effect and have decreased uncertainty by “borrowing strength” from all the other trials.

There was a one-to-one correlation between time of diet initiation and tumor location, as all experiments with intracranial tumors started the diet a few days after tumor manifestation and vice versa. Accordingly, in meta-regression both the tumor location and the time at which the KD was initiated were able to account for 26% of the heterogeneity between studies measuring a MR. Brain tumors and the switch to the KD later than the day of tumor initiation were associated with less effectiveness of the KD with more than 90% probability (*β*_late diet initiation_ = *β*_brain tumor_ = 0.29, 90% HPDI = [0.003, 0.54]). In this subgroup of studies the MR estimate was 0.89 (95% HPDI = [0.76, 1.04]), still supporting a positive effect, albeit no longer significantly.

The ketogenic ratio was able to explain only 1% of the heterogeneity between experiments measuring a MR. An increase in the ketogenic ratio by 1 was thereby associated with a slight decrease in the MR of -0.055 (95% HPDI = [-0.19, 0.08]), although only the 65% HPDI excluded a zero effect (65% HPDI = [-0.11,-5.7×10^−4^]). For tumor model (syngeneic/xenogeneic) or year of publication, no significant effects on the MR were found. None of the tested covariates were significantly correlated with a moderation of the overall HR, and accordingly no covariate was able to explain part of the heterogeneity between studies assessing a HR.

## Discussion

This meta-analysis indicates that in mice a KD prolongs survival (MR<1) and reduces the risk of experiencing the pre-defined endpoint (HR<1) compared to a high-carbohydrate SD when used as a monotherapy. It is therefore in line with a previous review by Lv et al. [9] in which eight of the nine included mouse studies showed a protective effect against cancer, although no systematic analysis of any outcome measure was conducted. We chose the MR as our primary outcome, since in most KD studies all animals experienced the pre-defined endpoint, so that the risk at the end of follow-up was not an issue [24].

The protective effect of the KD is most likely related to the state of ketosis, which was the most consistent covariate across studies. In particular, survival seems to be less dependent on weight loss in the KD group since most studies reported similar weight trends in both groups. Amongst several putative effect moderators only the time of KD initiation or alternatively tumor location were found to influence survival times and account for some of the between-study heterogeneity, as all brain tumor models included in the analysis for MR were also the ones using a late switch to the KD and vice versa. With more than 90% probability, the studies supported a survival-prolonging effect when the KD was started early (day of tumor cell injection) compared to at least one day after tumor cell injection or—alternatively—when a subcutaneous tumor instead of an intracranial one was used. Since it is currently not possible to differentiate both effects based on the studies evaluated in this meta-analysis, other evidence could be considered to reach a careful conclusion.

A protective role of the KD against early stages of tumorigenesis, but a much lesser effect when tumor growth has already been initiated, would be consistent with results from the largest rodent study on KD and cancer growth conducted to date. In this study, a total of 303 rats were used to investigate the effects of a carbohydrate-free diet started either before or concurrently with tumor transplantation [48]. These experiments strongly implied that a carbohydrate-free diet started several weeks before tumor transplantation “…produces such an influence upon the rats as to make them more resistant to tumor growth”, but also “…one is left in no doubt on the ineffectiveness of non-carbohydrate feeding begun at the time of tumor transplantation” [48]. Similarly, Moreschi’s seminal study from 1909 found a much stronger tumor growth inhibiting or even preventing effect when mice received a calorically restricted diet several days before rather than after tumor transplantation [1]. Finally, the meta-analysis by Lv et al. [9] revealed a strong protective effect of preventive calorie restriction against tumor incidence with a pooled odds ratio of 0.20 (95% CI [0.12,0.34]). Since the KD and calorie restriction share similar metabolic effects, the interpretation that timing of the KD rather than the tumor location matters would be consistent with these observations.

On the other hand, Seyfried and coworkers have argued that unrestricted KDs are not effective against various brain tumor models [18], so the role of tumor location as a moderator of survival times cannot be ruled out. Future studies should therefore assess the impact of an early versus late switch to a KD in brain tumor and subcutaneous models to differentiate the influence of both covariates.

Translated into the clinic, our result would imply at best a weak effect of KDs as the sole therapy against either already manifested tumors in general and/or brain tumors in particular. It is interesting that both hypotheses are consistent with the findings from human studies on glioblastoma multiforme, in which a KD as monotherapy seems ineffective in retarding tumor growth but more promising when combined with standard treatments [49]. It is important to stress that the experiments in which the switch to the KD occurred after tumor induction reflect more the clinical situation in which patients adopt the KD as a supportive therapy after being diagnosed with cancer. The situation of preventive timing of the KD may only apply to secondary prevention—when patients try to influence their risk of recurrence of an eradicated tumor. Therefore, in addition to testing the impact of tumor location and timing of the diet, future studies in which a KD is started together with standard cancer treatment are important as they probably have the greatest translational relevance. The results of the few such studies published so far are indeed promising [19,38].

Our meta-analysis has several limitations. First, all animal studies assessing the KD have a small sample size which leads to large uncertainties on the outcome measure. While this meta-analysis can therefore help to reach an overall conclusion with better precision, one must provide caution with definitive conclusions, especially as other non-random biases not accounted for can exist. Secondly, as expected, there was a moderate to large amount of heterogeneity present. Although tumor location and/or the time of KD initiation were able to account for roughly a quarter of this heterogeneity between studies using MR as the effect measure, much of it remains unexplained and probably relates to the large variety of mouse strains, tumor cell lines and endpoint criteria used. However, regardless the source of heterogeneity the results were highly robust against various a priori assumptions about the heterogeneity. Thirdly, the results might be sensitive to the various amounts of bias identified but also unidentified due to underreporting. We have shown that removing three studies with financial conflicts of interest still gave an overall protective effect of the KD, yet this was no longer significant. Thus we judge the uncertainties of the overall result as higher than estimated due to various forms of bias. Fourthly, the relations between individual blood glucose and ketone body levels on survival remain elusive. There is evidence for the importance of minimizing the ratio of glucose-to-ketone body concentrations for brain tumor control, which would indicate that additional calorie restriction could make KDs even more efficient [50]. However, there was insufficient reporting of these quantities in the text of the reviewed studies providing a form of attrition bias and showing a clear need for more detailed reporting of such important covariates together with outcome statistics in future rodent studies. Furthermore, even if the mean values would have been exactly known, regressing on mean values of animal characteristics would be prone to the so-called aggregation bias which occurs when the relation between study mean values and outcomes do not reflect the relation between individual values and individual outcomes [27]. Our best efforts to obtain crude estimates from the graphs provided in each study indicated that KD mice had on average blood glucose and ketone body concentrations around 7.0 mM and 1.6 mM, respectively, compared to 8.5 mM and 0.3 mM in the SD groups. While this range of ketosis is also realistic for humans on a KD, the high blood glucose levels would more reflect those of diabetic humans. Although many cancer patients also exhibit signs of insulin resistance, the translational relevance of this remains unclear. It points out a general limitation of mouse feeding studies since the metabolic response of mice to a certain diet can be age-, sex- and strain-dependent and very different from that of humans [51,52]. For example, contrary to humans, the widely used C57Bl/6 mice exhibit large metabolic disturbances when placed on a ketogenic diet if protein is not concurrently drastically restricted [51]. Having a 7–8 times higher basal metabolic rate than humans, mice are also much more sensitive to calorie restriction and intermittent fasting [53], so that any inter-study differences in feeding behavior caused by, e.g., different housing conditions or dietary constituents causing different grades of satiety, could additionally influence the outcome despite *ad libitum* food provision in all studies.

Finally we note that several of the identified biases and other aspects of the tumor models provide caution when extrapolating the results to humans. For instance, in some studies, tumor cells are injected subcutaneously in the mouse prior to assessment of growth, as opposed to their native organ location [22,23,42,45]. In these cases, large tumor sizes relative to the mouse can be reached that in our experience (CO and UK) are no longer responsive to the KD. Thus the KD must be started early to be effective. This may further hint to a benefit of the KD to delay or inhibit tumor initiation, or in these studies, tumor implantation, if not started too late. Of note, two studies that implanted syngeneic glioma cells into the brain revealed a benefit from the KD [19,43].

In conclusion, we found that the published data thus far indicate that a KD impedes tumor growth in mice. Our analysis reveals that the primary moderators of this effect may be the tumor location (brain/subcutaneous) and time of diet initiation. However, the strong correlation between these two covariates in the studies makes the exact mechanism elusive. Furthermore, all studies suffered from various biases and underreporting of methods whose influence on our result also remains elusive. Future studies should therefore improve methodological reporting and evaluate the effects of early versus late KD initiation for both subcutaneous and intracranial tumors. Also the translationally most relevant setting of a KD initiation concurrent with standard therapies after cancer manifestation should be more frequently investigated. If the timing of the KD is of major importance this would imply a role of fasting and KDs as a prevention strategy in humans, but only a supportive role during cancer treatment which is consistent with the current available human data. Further studies in humans to test these hypotheses are warranted.
